# Real-world effectiveness and satisfaction with intravenous eptinezumab treatment in patients with chronic migraine: REVIEW, an observational, multi-site, US-based study

**DOI:** 10.1186/s10194-024-01764-9

**Published:** 2024-04-25

**Authors:** Charles Argoff, Steven P. Herzog, Ryan M. Smith, Sameer V. Kotak, Liza Sopina, Yvonna Saltarska, Seema Soni-Brahmbhatt, Fawad A. Khan

**Affiliations:** 1 Albany Medical Center, 47 New Scotland Ave, 12208 Albany, NY USA; 2 Texas Neurology, Dallas, TX USA; 3 St. Luke’s Health System, Meridian, ID USA; 4 Yorker Health Corp., Glen Rock, NJ USA; 5 LS Consulting, Odense, Denmark; 6 Lundbeck LLC, Deerfield, IL USA; 7 The McCasland Family Comprehensive Headache Center, Ochsner Neurosciences Institute, New Orleans, LA USA; 8 University of Queensland-Ochsner Clinical School, New Orleans, LA USA

**Keywords:** Chronic migraine, Real-world, Eptinezumab, Preventive migraine treatment, Patient satisfaction

## Abstract

**Background:**

Despite recent advancements in migraine treatment, some patients continue to endure significant disease burden. Due to the controlled nature of randomized trials in migraine prevention, many real-world patients with comorbidities or prior exposure to certain therapies are excluded. Capturing evidence of the effectiveness of treatment in real-world clinical settings can further shape treatment paradigms. The objective of this study was to develop a comprehensive understanding of both patients’ and physicians’ real-world experiences with eptinezumab for chronic migraine (CM).

**Methods:**

REVIEW (Real-world EVidence and Insights into Experiences With eptinezumab) is an observational, multi-site (*n* = 4), US-based study designed to evaluate real-world experiences of patients treated with eptinezumab and their treating physicians. Patients were ≥ 18 years of age, with a diagnosis of CM, who had completed ≥ 2 consecutive eptinezumab infusion cycles (≥ 6 months of exposure). The study included a retrospective chart review, a patient survey, and a semi-structured physician interview that assessed patient and/or physician satisfaction with elements of daily living / well-being, migraine symptomology, and perspectives of the eptinezumab infusion experience.

**Results:**

Of the 94 patients enrolled, 83% (78/94) were female, the mean age was 49.2 years, and the mean time since migraine diagnosis was 15.4 years. Before eptinezumab treatment, patients experienced a mean of 8 self-reported “good” days/month, which increased to 18 after treatment. Most patients took, on average, ≥ 10 days/month of prescription and/or over-the-counter medication (81% [75/93] and 66% [61/93], respectively) to treat migraine attacks before eptinezumab treatment, which dropped to 26% (24/93) and 23% (21/93) following eptinezumab treatment. Prior to receiving eptinezumab, 62% (58/93) of patients indicated being at least slightly concerned about infusions; after eptinezumab infusion, this dropped to 14% (13/93). These patient survey findings were consistent with physician responses.

**Conclusion:**

This real-world evidence study demonstrated high overall satisfaction with the effectiveness of eptinezumab treatment for CM among most patients and their physicians.

**Supplementary Information:**

The online version contains supplementary material available at 10.1186/s10194-024-01764-9.

## Graphical Abstract

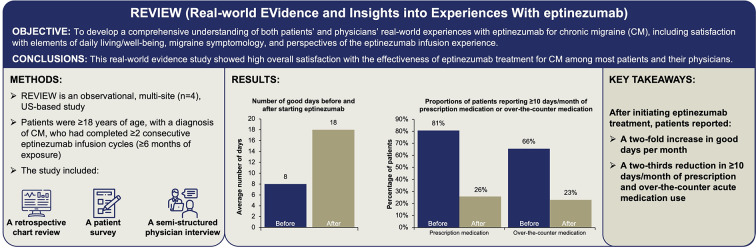

## Introduction

Migraine is a chronic disease affecting approximately 16% of adults [1, 2]. Chronic migraine (CM) is associated with a significant burden on school, work, and daily life [3–6]. Additionally, CM is associated with higher rates of certain comorbidities, such as anxiety and depression, higher disability, and a negative impact on relationships [5–7]. Traditionally, clinical trials assess impact of migraine therapies using objective measures, such as reduction in monthly migraine days, reduction in acute medication use, and impact on health-related quality of life, through validated outcome measures [8]. In the real-world setting it is important to not only recognize the limitations of using these validated tools, but also to consider the holistic burden on patients living with migraine. More research is needed to evaluate the holistic impact of migraine with patient-centric endpoints, inclusive of patients who are typically excluded from randomized, controlled clinical trials [9]. This underscores the importance of obtaining real-world data to inform clinical decision-making.

Many advanced preventive therapies for migraine have become available, including onabotulinumtoxinA [10] and a newer class of monoclonal antibodies (mAbs) that target the calcitonin gene-related peptide (CGRP) or its receptor [11]. This class of drugs includes eptinezumab [12], fremanezumab [13], galcanezumab [14], and erenumab [15]. Eptinezumab, a humanized immunoglobulin G1 (IgG1) mAb that binds the CGRP ligand and blocks its ability to bind to CGRP receptors, is indicated for the prevention of migraine in adults [12, 16]. One of the large-scale clinical trials (DELIVER) that evaluated the efficacy, safety, and tolerability of eptinezumab for migraine prevention included participants with migraine who had two to four previous preventive treatment failures; however, potential participants were not eligible if they had used advanced migraine therapies, including other anti-CGRP mAbs [17].

In real-world practice, given the availability of multiple advanced migraine therapies, it is likely that patients may have prior exposure to these advanced migraine therapies. So, while eptinezumab has proven effective in preventing migraine and increasing health-related quality of life in clinical trials in patients who are naïve to advanced migraine therapies [17–21], there is a need to explore the real-world experiences of eptinezumab use in patients with exposure to other advanced migraine therapies. Additionally, in the design of randomized clinical trials, there are limited opportunities to gather and analyze real-world clinician experiences and perspectives that could provide meaningful data to guide clinicians in their future prescribing decisions. The objective of the current study was to understand both patients’ and physicians’ real-world experiences with eptinezumab for migraine prevention in patients with CM.

## Methods

### Study design and participants

REVIEW (Real-world EVidence and Insights into Experiences With eptinezumab) was an observational, multi-site study in outpatients being treated with eptinezumab for CM. This study was intentionally conducted at four geographically dispersed study sites across the United States (Albany Medical Center, Albany, NY; Ochsner Medical Center, New Orleans, LA; Texas Neurology, Dallas, TX; and St. Luke’s Neurology, Meridian, ID).

REVIEW was composed of a structured patient survey (∼ 15–20 min to complete) evaluating patient perception on satisfaction and impact of their migraine treatment on migraine symptomology, various elements of daily living, including the eptinezumab infusion experience; a retrospective chart review to characterize demographics, medical history, and treatment history of the patients; and a semi-structured healthcare provider interview to assess the physicians’ satisfaction, experience, and treatment decision-making with eptinezumab. Study sites selected and recruited patients based on the inclusion/exclusion criteria outlined below. Patients who met the criteria were sorted on month of birth starting from January, and the first 25 from each site were selected to obtain consent for the patient self-report survey part of the study. Surveys were administered at the next infusion or office visit at three of the four sites, while one site administered patient surveys electronically after consent was obtained. Not all patients responded to each question; therefore, the base number of respondents may vary between questions. Prior to this study, a pilot linguistics validation study was conducted in 10 real-world patients with migraine (outside of the 94 included in this study) to assess contextual interpretation of survey questions. Appropriate adjustments to the question(s) format and response scales were made to maximize patient understanding of the intent of the question.

To be included in this study, patients must have been ≥ 18 years of age and with a diagnosis of CM (as indicated in the patient chart or adjudicated by the treating physician), must have completed ≥ 2 consecutive eptinezumab infusion cycles (equivalent to ≥ 6 months of exposure), must have been continuously followed at the study site for 6 months prior to the index date (defined as the date on which eptinezumab was first prescribed in a patient’s medical record), still be a patient at the site at the time of the study, and be able to complete the survey in English. Patients were excluded from this study if they were treated with eptinezumab in a clinical trial setting or were enrolled in a clinical trial for migraine or other headaches at the time of this study. Each study site obtained Institutional Review Board (IRB) approval (WCG IRB, Princeton, NJ, United States, and Ochsner Neurosciences Institute IRB, New Orleans, LA, United States) and all participants gave written informed consent prior to study participation.

### Study objectives

The key objective of this study was to assess the real-world effectiveness, infusion experience, and clinical impact of eptinezumab from both patient and physician perspectives. Multiple domains were evaluated in the patient self-report survey, including burden of migraine; treatment history; satisfaction with infusion experience; satisfaction with eptinezumab’s impact on reducing migraine frequency, severity, symptomology, including brain fog; acute medication use; and impact on productivity and several elements of daily living. Physician semi-structured interviews assessed reasons for initiating eptinezumab, satisfaction with effectiveness parameters (e.g., reduction in migraine/headache days, speed of onset), opinions on congruent domains listed above from the patient survey, and overall patient feedback on the infusion experience.

### Data collection and handling

Patient surveys were administered by study site monitors either in person during patients’ pre-scheduled visit at the site, or via secure electronic exchange between site and patient. If a study site permitted an external study monitor onsite, chart information was collected onsite; otherwise, chart reviews were conducted by study site personnel. Physician semi-structured interviews were conducted via virtual platforms and transcribed for thematic analysis. Study sites assigned patient study IDs to each recruited patient. All patient study files were anonymized (any name or other identifying information removed from data) and labeled with the patient study ID. All data provided or transferred to the external study monitor were stored on the secure Sponsor servers, in accordance with data safety regulations.

### Statistical analysis

All assessment data, including demographics, were summarized using descriptive techniques. Summary statistics (mean, standard deviation, median, minimum, and maximum values) are presented for continuous variables. Statistical analyses were based on patients with observed data. Counts and percentages are presented for categorical and binary variables. All analyses were conducted using MS Excel v10 and Stata BE (version 17, StataCorp, College Station, TX).

## Results

### Study population

A total of 94 patients from four study sites were included in this study. Patients were primarily female (83%, 78/94) and White or Caucasian (89%, 84/94), with a mean age of 49.2 years. On average, patients were diagnosed with migraine for 15.4 years, with many self-reporting comorbidity with psychiatric conditions (encompassing depression, anxiety, bipolar disorder, and other self-reported psychiatric conditions), allergies, cardiovascular conditions (including hypertension, heart disease, and heart attacks), and inflammatory conditions (such as rheumatoid arthritis, fibromyalgia, and other non-specified pain/inflammation) (Table 1). All patients (94/94) self-reported the use of another preventive therapy: 89% (84/94) reported prior subcutaneous anti-CGRP mAb use, 82% (77/94) reported onabotulinumtoxinA use, and 74% (70/94) reported use of an oral preventative. Of those who received at least one prior subcutaneous anti-CGRP mAb, 32% (27/84) reported switching once, 38% (32/84) reported switching twice, and 30% (25/84) reported switching subcutaneous anti-CGRP mAb therapies three times. The use of a gepant (acute or preventive usage) was reported by 73% of patients (69/94). Of those who received at least one gepant, 51% (35/69) reported receiving one, 36% (25/69) reported receiving two, and 13% (9/69) reported receiving three.

**Table 1:** Patient-reported demographics and clinical characteristics

	Patients *N* = 94
**Gender, n (%)**	
Female	78 (83)
Male	14 (15)
Non-binary	2 (2)
**Mean (median) age, years**	49.2 (49)
**Race, n (%)**	
White or Caucasian	84 (89)
Black or African American	8 (9)
Asian	1 (1)
Other	1 (1)
**Ethnicity, n (%)**	
Non-Hispanic or -Latino	89 (95)
Hispanic or Latino	5 (5)
**Mean (median) years since migraine diagnosis**	15.4 (12)
**Primary insurance type**	
Commercial/employer	47 (50)
Medicare	35 (37)
Medicaid	6 (6)
Other	6 (6)
**Comorbidities, n (%)**	
Psychiatric conditions	61 (65)
Anxiety	47 (50)
Depression	45 (48)
Bipolar disorder	3 (3)
Other	4 (4)
Allergies	53 (56)
Cardiovascular disease	38 (40)
Hypertension or high blood pressure	35 (37)
Heart attack	2 (2)
Heart disease	1 (1)
Inflammatory conditions	37 (39)
Fibromyalgia	16 (17)
Rheumatoid arthritis	11 (12)
Other pain/inflammation	26 (28)
Digestive system conditions	36 (38)
Sleep apnea	25 (27)
Asthma	15 (16)
**Number of eptinezumab infusions received, n (%)**	
1^a^	1 (1)
2	12 (13)
3	15 (16)
4	17 (18)
5 or more	48 (52)
Missing	1 (1)
**Current eptinezumab dose received, n (%)**	
100 mg	22 (23)
300 mg	54 (57)
Other	2 (2)
Cannot recall	13 (14)
Missing	3 (3)
**Previously received another preventive therapy, n (%)**	94 (100)
**Previous therapy types, n (%)** ^**b**^	
Prior subcutaneous anti-CGRP mAb	84 (89)
OnabotulinumtoxinA	77 (82)
Oral preventive	70 (74)
Gepant^c^	69 (73)

^a^One patient self-reported only receiving 1 eptinezumab infusion: however, all patients included in the study had ≥ 2 infusions, confirmed by the physician and chart review

^b^Concomitant use of therapies post-eptinezumab initiation is unknown

^c^Includes atogepant, ubrogepant, and rimegepant; therefore, captures both acute and preventive use

CGRP, calcitonin gene-related peptide; mAb, monoclonal antibody

### Patient experience with eptinezumab for migraine prevention

**Fig. 1:**
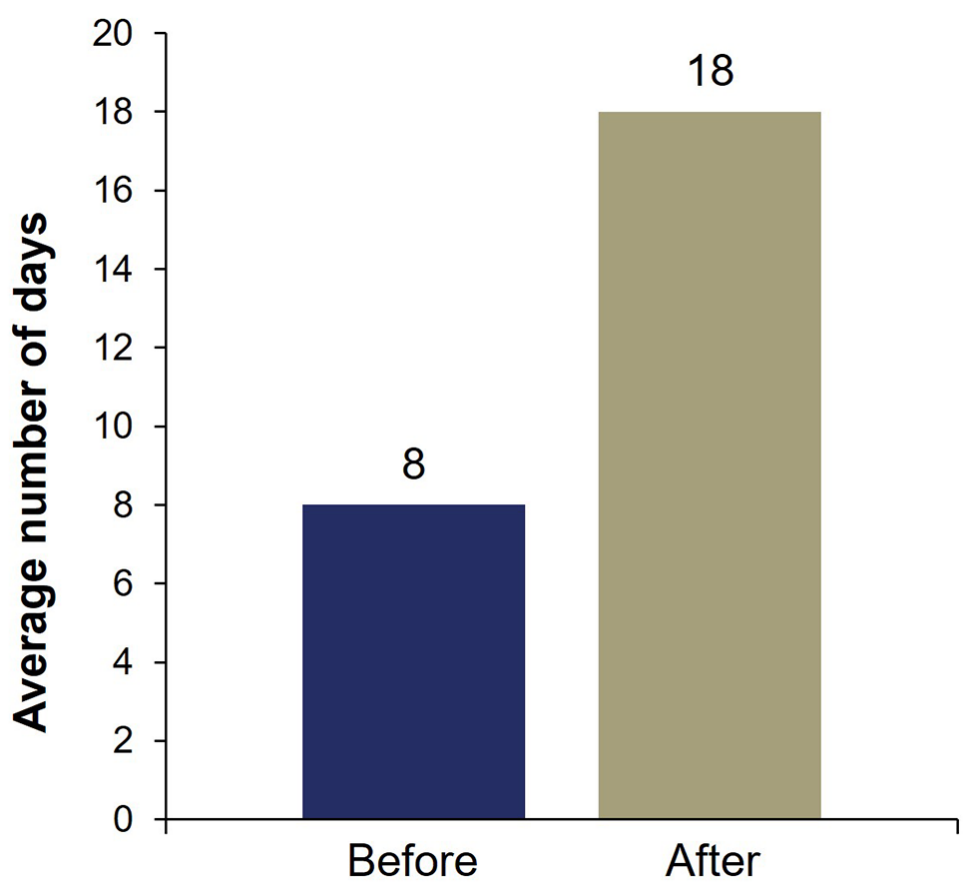
Patient-reported average number of good days/month before and after starting eptinezumab Patients were asked, “On average, how many good days per month did you experience before/after starting eptinezumab? Please indicate the number of days from 1‒31.”

Approximately half of patients (52%, 48/93) self-reported they had received ≥ 5 eptinezumab infusions (≥ 15 months of eptinezumab treatment). Per chart review data, the mean (median) total number of infusions received for the total study population (*N* = 94) was 4.5 (4.0); 51% (48/94) of patients started with eptinezumab 100 mg and transitioned to 300 mg, 1% (1/94) started on eptinezumab 200 mg (not a Food and Drug Administration [FDA] approved dose) and transitioned to 300 mg, and 48% (45/94) did not undergo any dosing changes (i.e., remained at eptinezumab 100 mg, 200 mg, or 300 mg based on starting dose). Before starting eptinezumab, the mean number of self-reported “good days” per month was 8 days and it more than doubled, to 18 days, following eptinezumab treatment (median “good days” were 7 days before and 20 days after eptinezumab initiation) (Fig. 1).

### Patient-reported impact on acute medication use and migraine symptomology

**Fig. 2:**
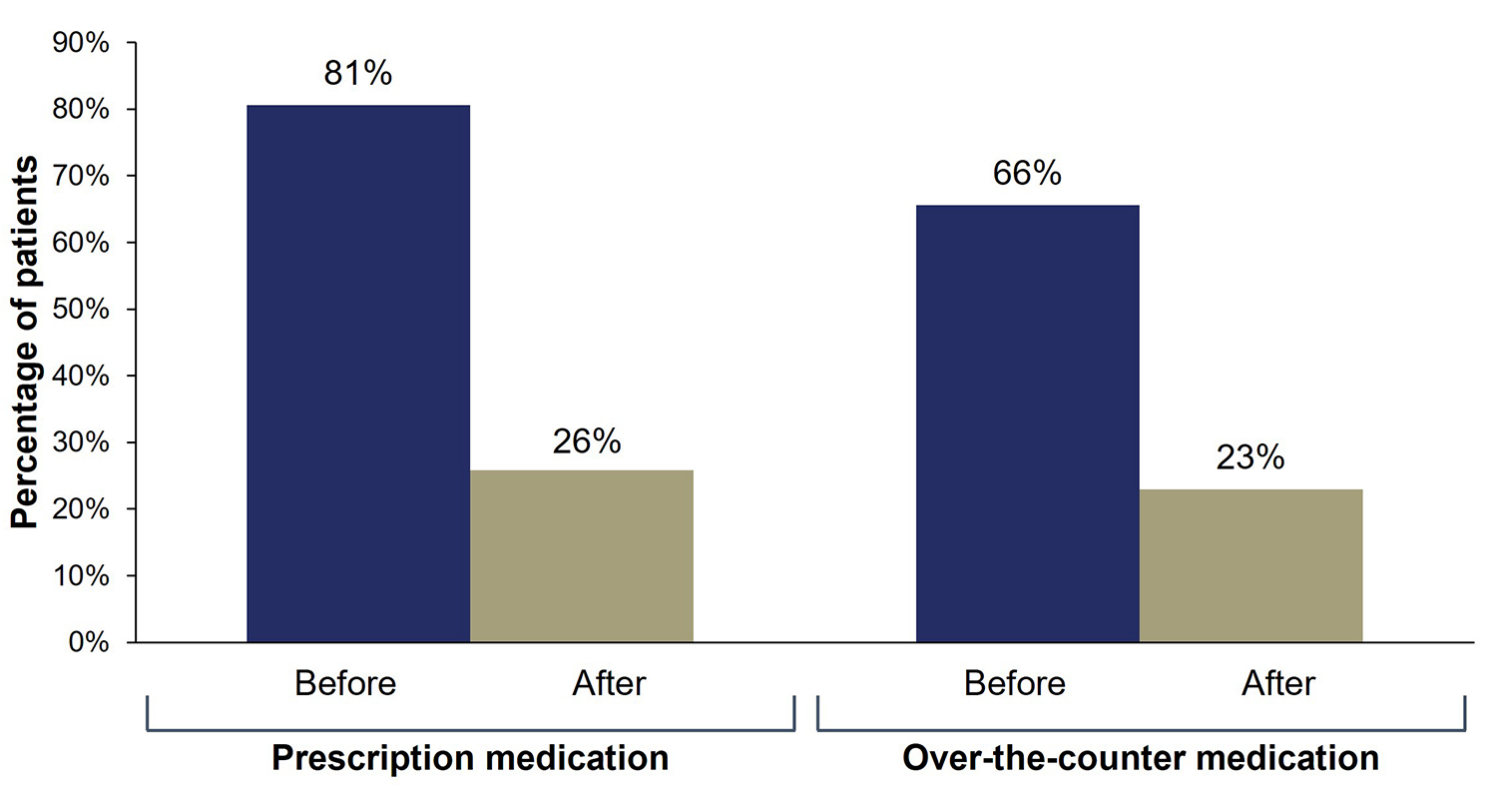
Percentage of patients reporting ≥ 10 acute medication days/month before and after starting eptinezumab Patients were asked: “How many days per month, on average, did you take prescribed / over-the-counter medications to treat your migraine attacks once they started? (In the 3 months before starting eptinezumab and after starting eptinezumab).” Choices included: 0, 1‒4, 5‒9, and 10 + days.

Before starting eptinezumab treatment, 81% (75/93) of patients reported using ≥ 10 days/month of prescription acute medication. After starting eptinezumab treatment, this number reduced to 26% (24/93). Similarly, before eptinezumab treatment 66% (61/93) reported ≥ 10 days/month of over-the-counter acute medication, and after starting eptinezumab treatment, this proportion of patients was reduced to 23% (21/93) (Fig. 2, Supplemental Table 1).

**Fig. 3:**
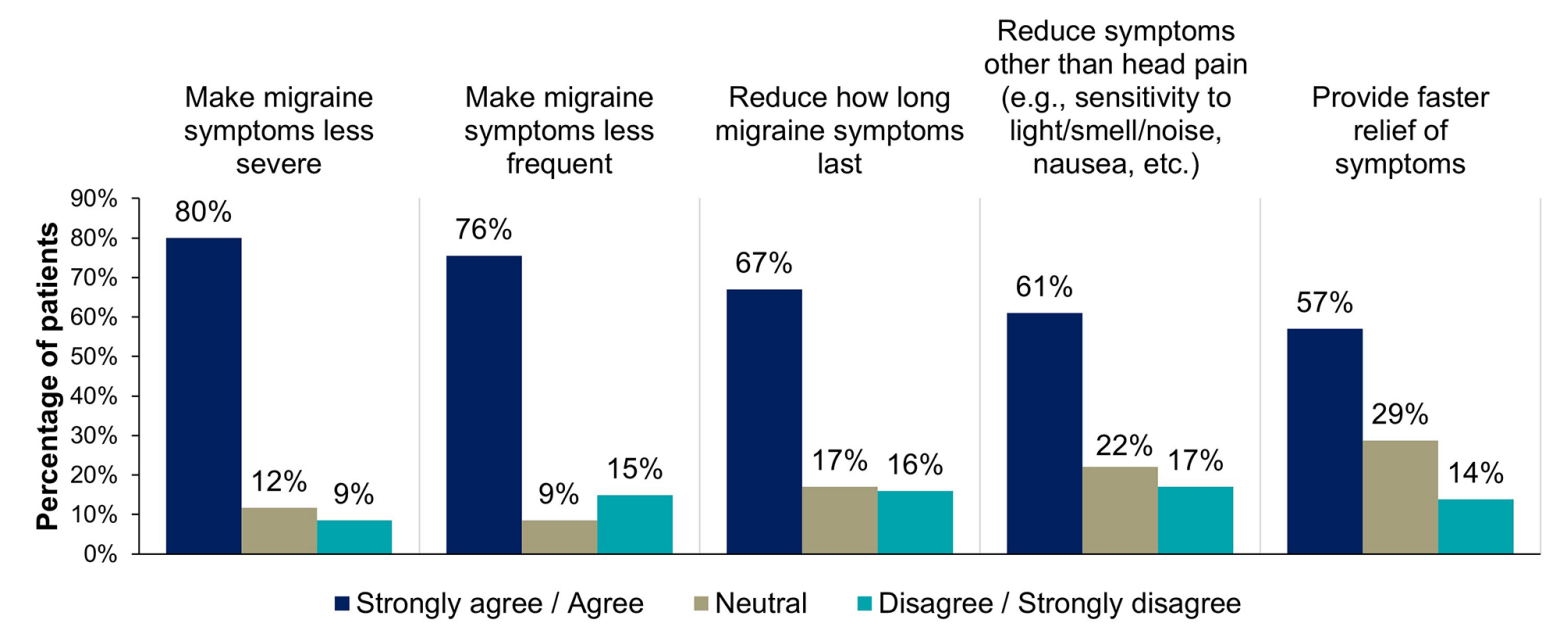
Patient-reported satisfaction with eptinezumab’s ability to impact migraine symptoms Percentages may not add up to 100% due to rounding. Patients were prompted: “Please rate how much you agree or disagree with the following statements by placing a checkmark ✓ in the column which most closely fits your opinion: I am satisfied with eptinezumab’s ability to…” Choices included: strongly agree, agree, neutral, disagree, and strongly disagree.

**Fig. 4:**
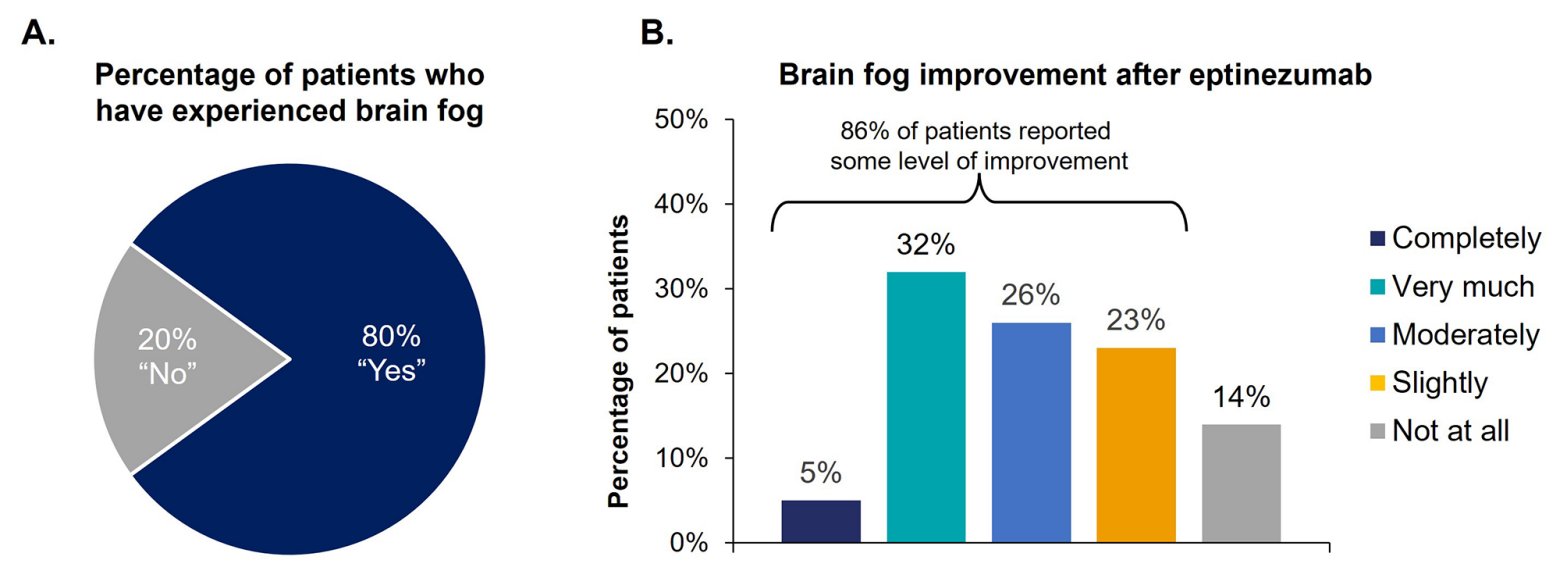
Percentage of patients who reported experiencing brain fog (**A**) and improvement after eptinezumab treatment (**B**) Percentages may not add up to 100% due to rounding. Patients were asked: “Have you experienced ‘brain fog’ (feeling confused, have difficulty learning or remembering, or have trouble speaking or reading)? If yes, please rate to what extent your symptoms have improved since starting eptinezumab.” Choices included: completely, very much, moderately, slightly, or not at all.

Before starting eptinezumab, the migraine-related symptoms most commonly rated as very or extremely bothersome to patients were head pain (95%, 89/94) and head pain that worsened with any movement or routine physical activity (88%, 81/92), followed by difficulty concentrating or thinking clearly (78%, 73/94) and sensitivity to light (68%, 64/94) (Supplemental Fig. 1). A higher percentage of patients reported that they agreed/strongly agreed with eptinezumab’s ability to impact their migraine symptomology (e.g., severity, frequency, onset of relief) (Fig. 3, Supplemental Table 2). Eighty percent (74/93) of patients reported they had experienced “brain fog” (i.e., feeling confused, have difficulty learning or remembering, or have trouble speaking or reading) (Fig. 4A, Supplemental Table 3), and 86% (64/74) of these patients reported their brain fog symptoms improved to some degree (slightly, moderately, very much, or completely) after treatment with eptinezumab (Fig. 4B, Supplemental Table 3).

### Patient-reported impact on health-related quality of life and treatment goals

**Fig. 5:**
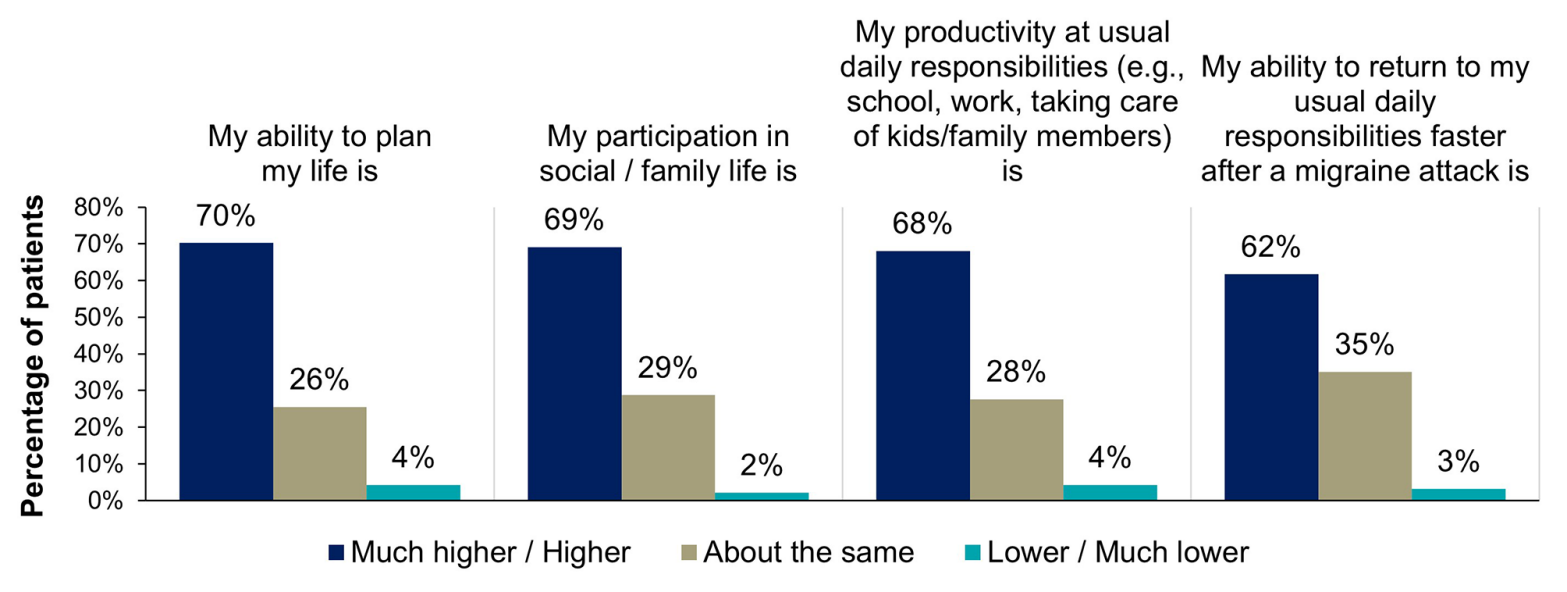
Patient-reported impact on elements of daily living since starting eptinezumab Percentages may not add up to 100% due to rounding. Patients were prompted: “Please rate the following statements on different aspects of your life (i.e., your feelings) by placing a checkmark ✓ in the column which most closely fits your opinion: After starting eptinezumab, my satisfaction with…” Choices included: much higher, higher, about the same, lower, and much lower.

**Fig. 6:**
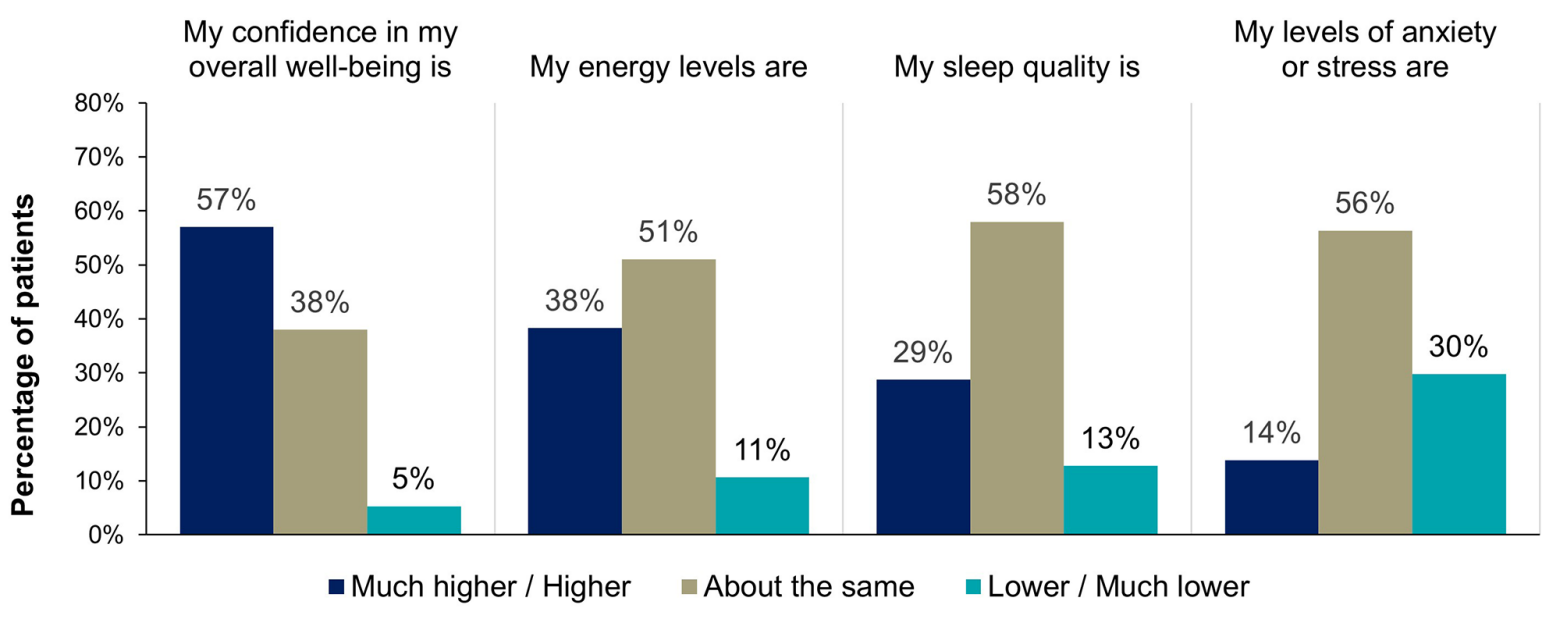
Patient-reported impact on elements of well-being since starting eptinezumab Percentages may not add up to 100% due to rounding. Patients were instructed: “Please rate the following statements on different aspects of your life (i.e., your feelings) by placing a checkmark ✓ in the column which most closely fits your opinion: After starting eptinezumab, my satisfaction with…” Choices included: much higher, higher, about the same, lower, and much lower.

After starting eptinezumab treatment, the majority of patients reported higher or much higher satisfaction with various elements of daily living, including ability to plan their life (70%, 66/94), participation in social/family life (69%, 65/94), productivity at usual daily responsibilities (68%, 64/94), and ability to return to daily responsibility faster (62%, 58/94) (Fig. 5, Supplemental Table 4). After starting eptinezumab treatment, 57% (53/93) of patients reported higher or much higher confidence in their overall well-being. Less change was reported in the other domains of well-being, including energy levels, sleep quality, and anxiety/stress levels (Fig. 6, Supplemental Table 4).

Of the 94 patients who completed the survey, more than half (59%, 55/94) of patients reported setting treatment goals for migraine with their physician, with 26% (24/94) not setting goals and 16% (15/94) unsure if they set goals. Of those who set treatment goals in collaboration with their physician, 56% (31/55) separately reported achieving their treatment goals (individual and/or in collaboration with their physician). Of patients who did not set treatment goals with their physician, 33% (8/24) reported achieving their treatment goals.

### Infusion experience

**Fig. 7:**
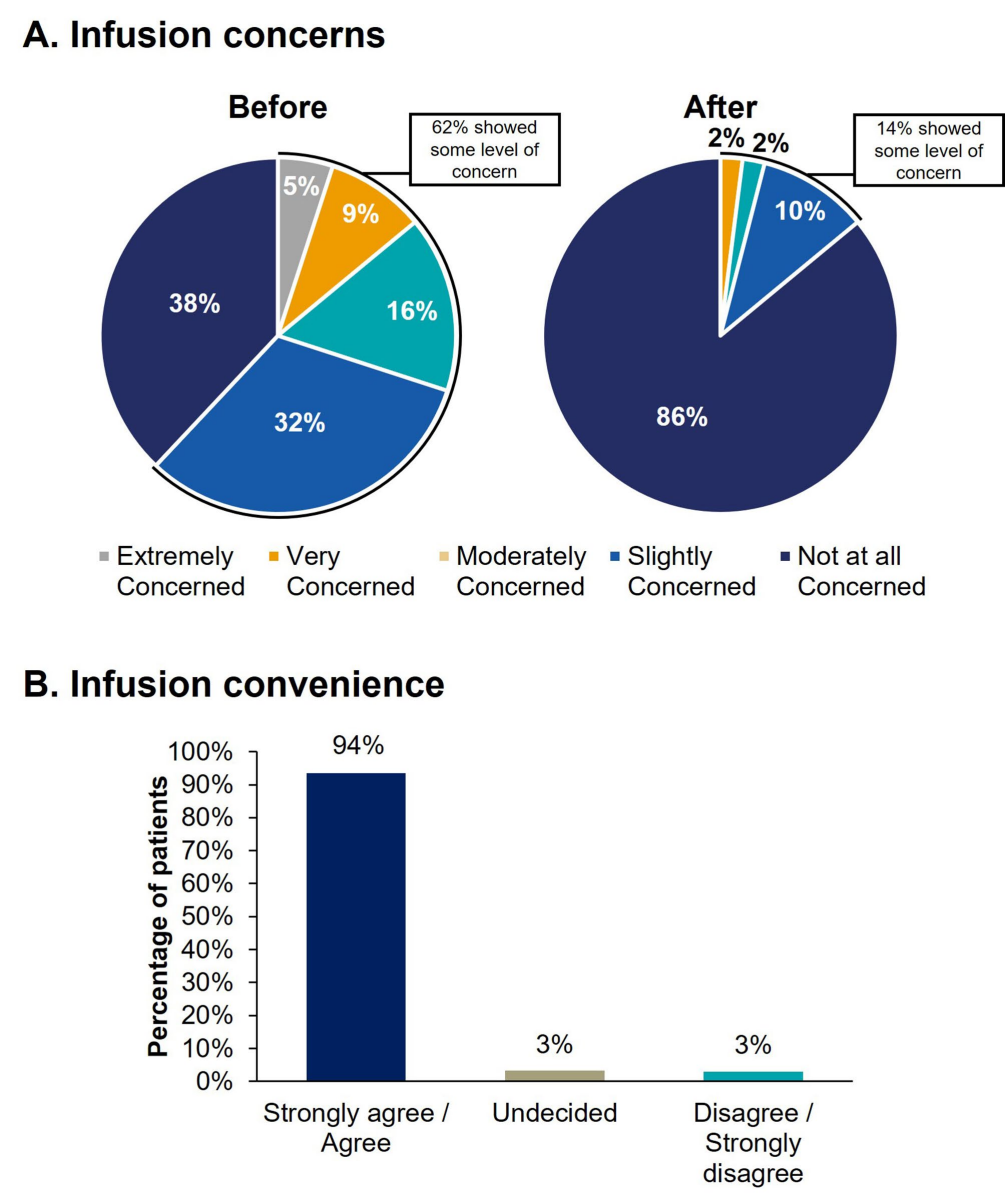
Infusion concerns before and after eptinezumab treatment (**A**) and convenience of treatment through infusion (**B**) Percentages may not add up to 100% due to rounding. Patients were instructed: “Please rate how much you agree or disagree with the following statements by placing a checkmark ✓ in the column which most closely fits your opinion.” (A) I had concerns about receiving infusions; choices included: extremely concerned, very concerned, moderately concerned, slightly concerned, and not at all concerned. (B) I find it convenient to receive my treatment through an infusion; choices included: strongly agree, agree, undecided, disagree, and strongly disagree.

Prior to eptinezumab infusion, 62% (58/93) of patients indicated being at least slightly concerned about receiving an infusion; after eptinezumab infusion, this decreased to 14% (13/93) (Fig. 7A, Supplemental Table 5A). Moreover, 94% (87/93) of patients agreed or strongly agreed that it was convenient to receive treatment via an intravenous infusion (Fig. 7B, Supplemental Table 5B). Overall physician feedback received from patients on the eptinezumab infusion experience was also positive or very positive (median score of 4.8 on a 5-point scale where 1 indicated “very negative” and 5 indicated “very positive”).

### Clinician perspective

Overall, data from the prescribing physicians’ semi-structured interviews supported patient-reported data. Physicians indicated that patients’ utilization of acute headache/migraine medication decreased following initiation of eptinezumab treatment (median score of 5.0 on a 5-point scale, where 1 indicated a “significant increase” in acute medication utilization and 5 indicated a “significant decrease” in utilization after initiation of eptinezumab treatment). The physicians corroborated patient impressions, with physicians reporting high levels of satisfaction with eptinezumab’s speed of onset, ability to reduce monthly migraine/headache days, impact on participation in social/family life, and impact on patient productivity (median scores were 4.5, 5.0, 4.5, and 4.5, respectively, on a 5-point scale where 1 indicated “very dissatisfied” and 5 indicated “very satisfied”).

## Discussion

Although several controlled clinical trials have established the efficacy and safety of eptinezumab [17–21], this study examined the effectiveness and satisfaction of eptinezumab in a real-world clinical setting of 94 patients with CM from multiple headache centers in the United States. In contrast to the participant populations in the eptinezumab clinical trials, this study included a broader range of patients with comorbidities, and patients with prior exposure to newer migraine-specific preventive therapies, including anti-CGRP mAbs, onabotulinumtoxinA, and gepants, thus more accurately reflecting current real-world patient characteristics.

Data from this study indicate that, despite prior exposure to various preventive therapies, patients self-reported a positive impact on a variety of domains after initiating eptinezumab treatment. Moreover, patients reported a higher degree of confidence in their overall well-being. Remarkably, this study demonstrated that patients experiencing continued migraine burden who had previously tried other anti-CGRP mAb preventive treatments responded positively to eptinezumab; this suggests empirically that exposure and trial of one anti-CGRP mAb does not necessarily preclude a positive response to eptinezumab treatment. Recent studies also corroborate this finding [22–25], with one study showing that patients with previous exposure to erenumab or galcanezumab who subsequently initiated eptinezumab treatment had an overall reduction in monthly migraine days of ∼ 8.4 and ∼ 8.2, respectively, over a 6-month period [22]. These findings have implications for public health and best practices for clinicians navigating through treatment decisions among a variety of advanced migraine therapies. They substantiate the existence of human-to-human variability in responsiveness to anti-CGRP mAbs. They also suggest that general policies from payors and government health plans at both local and national levels, which dictate the selection of some treatments while not offering others, are not fully supported by the most current evidence [26].

Patients reported that their number of monthly “good” days doubled after commencing eptinezumab treatment. This is important, as the definition of “good days” was defined by the individual patient. Additionally, this outcome measure shifts the focus to a patient feeling well compared to other measures which solely focus on when a patient feels poorly. These findings underscore a substantial improvement in patients’ perception of overall well-being through eptinezumab treatment. This is especially noteworthy in a subpopulation with a history of trying several preventive therapies for CM. Illness perception has been demonstrated to influence factors such as chronicity, quality of life, treatment adherence, and psychosocial responses in various diseases, including those with a high burden of headache. In the case of CM, this connection is particularly relevant, as it correlates with a lower quality of life. Therefore, this self-reported measure suggests that positively influencing illness perception in CM may serve as a proxy for improved quality of life and reducing the burden of the disease.

Patients experiencing a high frequency of monthly migraine days often rely on frequent use of acute headache medication, which can lead to medication overuse [27–29]. Acute medication overuse is associated with many negative outcomes, including the risk of migraine chronification (progression from episodic migraine to chronic migraine) and medication-overuse headache [30]. In this study, patients, supported by the physicians’ report, reported a decrease in the use of acute migraine medications after initiating treatment with eptinezumab.

It is well known that migraine symptoms can vary from attack to attack and across individual patients. In this study, patients treated with eptinezumab reported high satisfaction with its ability to impact migraine symptomology. Offering patients a therapeutic option that effectively addresses their individualized symptoms can improve adherence, reduce or prevent the frequent use of acute medication, and ultimately lead to higher patient satisfaction.

Migraine-related comorbidities and burden can significantly affect various aspects of a patient’s life [31]. As a result, it is valuable to assess the disease-related burden beyond the impacts of migraine symptoms alone. In this study, questions were posed to explore theses effects on daily living. After initiating eptinezumab, patients reported higher satisfaction with elements of daily living, such as planning life and participation in social/family life and overall well-being. No treatment-related change and/or no worsening was observed in domains, including energy levels, sleep quality, and anxiety/stress levels. Patients may have comorbidities or social determinants of health that can affect these aspects of overall well-being, and these factors may be independent of the specific impact on migraine burden.

Brain fog, often associated with migraine, can have a debilitating impact on cognition and may occur during or between migraine attacks [32, 33]. The ability of migraine-specific therapies to improve brain fog has rarely been studied in clinical trials [18, 19, 34–36]. In this population of patients with CM, 80% had reported experiencing brain fog (i.e., feeling confused, difficulty learning or remembering, or trouble speaking or reading), indicating the pervasiveness of this symptom in this population. Remarkably, of the patients who said they had experienced brain fog, a large majority (86%) reported that they experienced some level of improvement in their brain fog after initiating eptinezumab treatment. To date, research evaluating the impact of preventive treatments on cognition are limited, with focus on traditional oral therapies and onabotulinumtoxinA [37]. These findings suggest that further research is needed to fully understand cognitive burden and the impact of preventive therapies.

A previous patient preference study evaluated attributes of non-oral preventive migraine medications that are most important to individuals with migraine. It was shown that individuals preferred a treatment with a quick speed of onset that lasted for its full treatment duration. Additionally, the previous study showed that while preference for the mode of administration varied between people, 75% of individuals did not think intravenous infusion was a barrier to care [38]. The REVIEW study corroborates the previous patient preference study results in that while 62% of patients indicated being at least slightly concerned about infusion before receiving eptinezumab, this number decreased to 14% following treatment. This indicates that a majority of patients experienced an improved acceptance of infusion as a route of administration. Moreover, almost all patients (94%) who received eptinezumab agreed that it was convenient to receive treatment via an infusion.

Before beginning a new treatment, patients can establish treatment goals either independently or in collaboration with their clinicians. The process of setting treatment goals represents a form of shared decision-making, facilitating better understanding of patients’ individual health needs and treatment preferences. This, in turn, leads to the development of personalized treatment plans. This study identified three categories of treatment goals: those related to symptom resolution, treatment approaches, and overall quality of life. Notably, only 59% of patients in this survey reported setting goals with their physicians. Therefore, ensuring alignment of treatment expectations between patients and prescribing physicians may be an area in need of improvement. Despite not all patients explicitly reporting the creation of treatment goals in collaboration with their physicians, 52% of the surveyed patients with CM affirmed that they successfully achieved their treatment goals while on eptinezumab, reflecting real-life improvements in their personal treatment objectives.

### Limitations

Patient data were survey-based and self-reported, thus dependent upon patient recall. Moreover, 51% of respondents reported receiving five or more infusions, which further impacts recall, but may reflect a patient population that has continued therapy due to positive response and/or good tolerability. One patient reported only receiving one infusion of eptinezumab; however, it was confirmed by investigators that all included patients had received at least two infusions. Gepants captured included atogepant, ubrogepant, and rimegepant; given the nature of our methodology, however, differentiation between preventive use versus acute use for rimegepant was not possible. In addition, concomitant use of therapies in conjunction with eptinezumab was not deciphered within the patient charts or patient survey. Moreover, it was not determined why patients switched therapies; it could be due to tolerability, efficacy, and/or payor-related reasons. No cross-comparisons were conducted among the outcome measures for the 100-mg, 200-mg, or 300-mg doses, and ad hoc analyses were not performed for patients transitioning between lower and higher doses. Further exploration of the specific reasoning and considerations for the 200-mg, an off-label eptinezumab dose, was not conducted. The final survey was provided to the patient with no additional guidance/interpretation; however, a linguistic assessment was performed in 10 patients to optimize survey questions prior to the start of the study. Patient diary data were unavailable, which limited further corroboration of effectiveness on a continual log basis, as opposed to cross-sectional patient recall. Moreover, inherent selection bias in this study could not be mitigated.

## Conclusions

The population studied here represented a group of individuals with CM who continued to experience migraine-related burden despite prior treatment with several preventive therapies. Notably, 89% of the participants had previously been treated with subcutaneous anti-CGRP mAbs, and many had tried various other advanced migraine preventive options available in the United States. Surprisingly, despite this history of prior treatment use, after initiating eptinezumab treatment, patients reported on average a two-fold increase in good days per month, along with a two-thirds reduction in patients with ≥ 10 days/month of prescription and over-the-counter acute medication use. Overall, most patients expressed satisfaction with the effect of eptinezumab in reducing the severity, frequency, and duration of their migraine symptoms. These real-world data findings, combined with positive physician and patient-reported experiences, offer valuable insights and clinically relevant information regarding the use of eptinezumab in the management of CM. This data can assist clinicians in making informed management decisions with the goal of improving patient outcomes.

### Electronic supplementary material

Below is the link to the electronic supplementary material.

Supplementary Material 1

Supplementary Material 2

## Data Availability

The data sets generated and/or analyzed during the current study are not publicly available due to data license restrictions. However, summarized data are available from the corresponding author upon reasonable request.

## Abbreviations

CGRP: Calcitonin gene-related peptide
CM: Chronic migraine
IgG1: Immunoglobulin G1
IRB: Institutional Review Board
mAb: Monoclonal antibody
REVIEW: **R**eal-world **EV**idence and **I**nsights into **E**xperiences **W**ith eptinezumab
