# Supplementary material for: Real-world effectiveness and satisfaction with intravenous eptinezumab treatment in patients with chronic migraine: REVIEW, an observational, multi-site, US-based study
Source: J Headache Pain. 2024 Apr 25;25(1):65. doi: 10.1186/s10194-024-01764-9 (PMC11044317; doi:10.1186/s10194-024-01764-9)
Supplement: Supplementary file 1 — Supplementary Material 1 [file 10194_2024_1764_MOESM1_ESM.pdf]

Supplemental Material

Supplemental Table 1. Patient-reported average days per month with prescribed or over-the-counter medication to treat migraine attacks before and after starting eptinezumab treatment

	*In the 3 months **before** starting eptinezumab*	***After** starting treatment with eptinezumab*
Prescription		
0 days	4 (4/93)	10 (9/93)
1–4 days	3 (3/93)	30 (28/93)
5–9 days	12 (11/93)	34 (32/93)
10 or more days	81 (75/93)	26 (24/93)
Over-the-counter		
0 days	17 (16/93)	28 (26/93)
1–4 days	9 (8/93)	28 (26/93)
5–9 days	9 (8/93)	22 (20/93)
10 or more days	66 (61/93)	23 (21/93)

Values are % (n/N). Percentages may not add up to 100 because of rounding. Patients were asked: “How many days per month, on average, did you take prescribed / over-the-counter medications to treat your migraine attacks once they started? (In the 3 months before starting eptinezumab and after starting eptinezumab).” Choices included: 0, 1–4, 5–9, and 10+ days.

Supplemental Table 2. Patient-reported satisfaction with eptinezumab's ability to impact migraine symptoms

	**Strongly agree**	**Agree**	**Neutral**	**Disagree**	**Strongly disagree**
I am satisfied with eptinezumab's ability to:					
…make migraine symptoms less severe	47 (44/93)	32 (30/93)	12 (11/93)	2 (2/93)	6 (6/93)
...provide faster relief of symptoms	35 (33/93)	22 (20/93)	29 (27/93)	6 (6/93)	8 (7/93)
...make migraine symptoms less frequent	45 (42/93)	31 (29/93)	9 (8/93)	6 (6/93)	9 (8/93)
...reduce how long migraine symptoms last	34 (32/93)	32 (30/93)	17 (16/93)	10 (9/93)	6 (6/93)
…reduce symptoms other than head pain (e.g., sensitivity to light/smell/noise, nausea, etc.)	34 (31/92)	27 (25/92)	22 (20/92)	10 (9/92)	8 (7/92)

Values are % (n/N). Percentages may not add up to 100% due to rounding. Patients were instructed: "Please rate how much you agree or disagree with the following statements by placing a checkmark ✓ in the column which most closely fits your opinion."

Supplemental Table 3. Patient-reported experience with brain fog and impact of eptinezumab

<table>
<tr><th></th><th colspan="5">Yes</th><th colspan="5">No</th></tr>
<tr><td>Have you experienced "brain fog" (feeling confused, have difficulty learning or remembering, or have trouble speaking or reading)…</td><td colspan="5">80 (74/93)</td><td colspan="5">20 (19/93)</td></tr>
<tr><td></td><td colspan="2">Completely</td><td colspan="2">Very Much</td><td colspan="2">Moderately</td><td colspan="2">Slightly</td><td colspan="2">Not at all</td></tr>
<tr><td>…if yes, please rate to what extent your symptoms have improved since starting Vyepti</td><td colspan="2">5 (4/74)</td><td colspan="2">32 (24/74)</td><td colspan="2">26 (19/74)</td><td colspan="2">23 (17/74)</td><td colspan="2">14 (10/74)</td></tr>
</table>

Values are % (n/N). Percentages may not add up to 100% due to rounding.

Supplemental Table 4. Patient-reported impact on elements of daily living and well-being since starting eptinezumab

	**Much higher**	**Higher**	**About the same**	**Lower**	**Much lower**
After starting eptinezumab, my satisfaction with…					
My ability to plan my life is	31 (29/94)	39 (37/94)	26 (24/94)	3 (3/94)	1 (1/94)
My participation in social and family life is	35 (33/94)	34 (32/94)	29 (27/94)	1 (1/94)	1 (1/94)
My productivity at usual daily responsibilities (e.g., school, work, taking care of kids/family members) is	32 (30/94)	36 (34/94)	28 (26/94)	1 (1/94)	3 (3/94)
My ability to return to my usual daily responsibilities faster after a migraine attack is	31 (29/94)	31 (29/94)	35 (33/94)	2 (2/94)	1 (1/94)
My energy levels are	17 (16/94)	21 (20/94)	51 (48/94)	10 (9/94)	1 (1/94)
My levels of anxiety or stress are	4 (4/94)	10 (9/94)	56 (53/94)	16 (15/94)	14 (13/94)
My sleep quality is	10 (9/93)	19 (18/93)	58 (54/93)	12 (11/93)	1 (1/93)
My confidence in my overall well-being is	24 (22/93)	33 (31/93)	38 (35/93)	5 (5/93)	0 (0/93)

Values are % (n/N). Percentages may not add up to 100% due to rounding. Patients were instructed: "Please rate the following statements on different aspects of your life (i.e., your feelings) by placing a checkmark ✓ in the column which most closely fits your opinion."

Supplemental Table 5. Patient-reported level of infusion concerns before and after eptinezumab treatment (A) and patient-reported infusion convenience (B)

A.

	**Extremely concerned**	**Very concerned**	**Moderately concerned**	**Slightly concerned**	**Not at all concerned**
I had concerns about receiving infusions:					
BEFORE *starting my treatment with eptinezumab*	5 (5/93)	9 (8/93)	16 (15/93)	32 (30/93)	38 (35/93)
AFTER *starting my treatment with eptinezumab*	0 (0/93)	2 (2/93)	2 (2/93)	10 (9/93)	86 (80/93)

B.

	**Strongly agree**	**Agree**	**Undecided**	**Disagree**	**Strongly disagree**
I find it convenient to receive my treatment through an infusion	60 (56/93)	33 (31/93)	3 (3/93)	2 (2/93)	1 (1/93)

Values are % (n/N). Percentages may not add up to 100% due to rounding. Patients were instructed: "Please rate how much you agree or disagree with the following statements by placing a checkmark ✓ in the column which most closely fits your opinion."

Supplemental Figure 1. Patient-reported bothersomeness of symptoms before starting eptinezumab

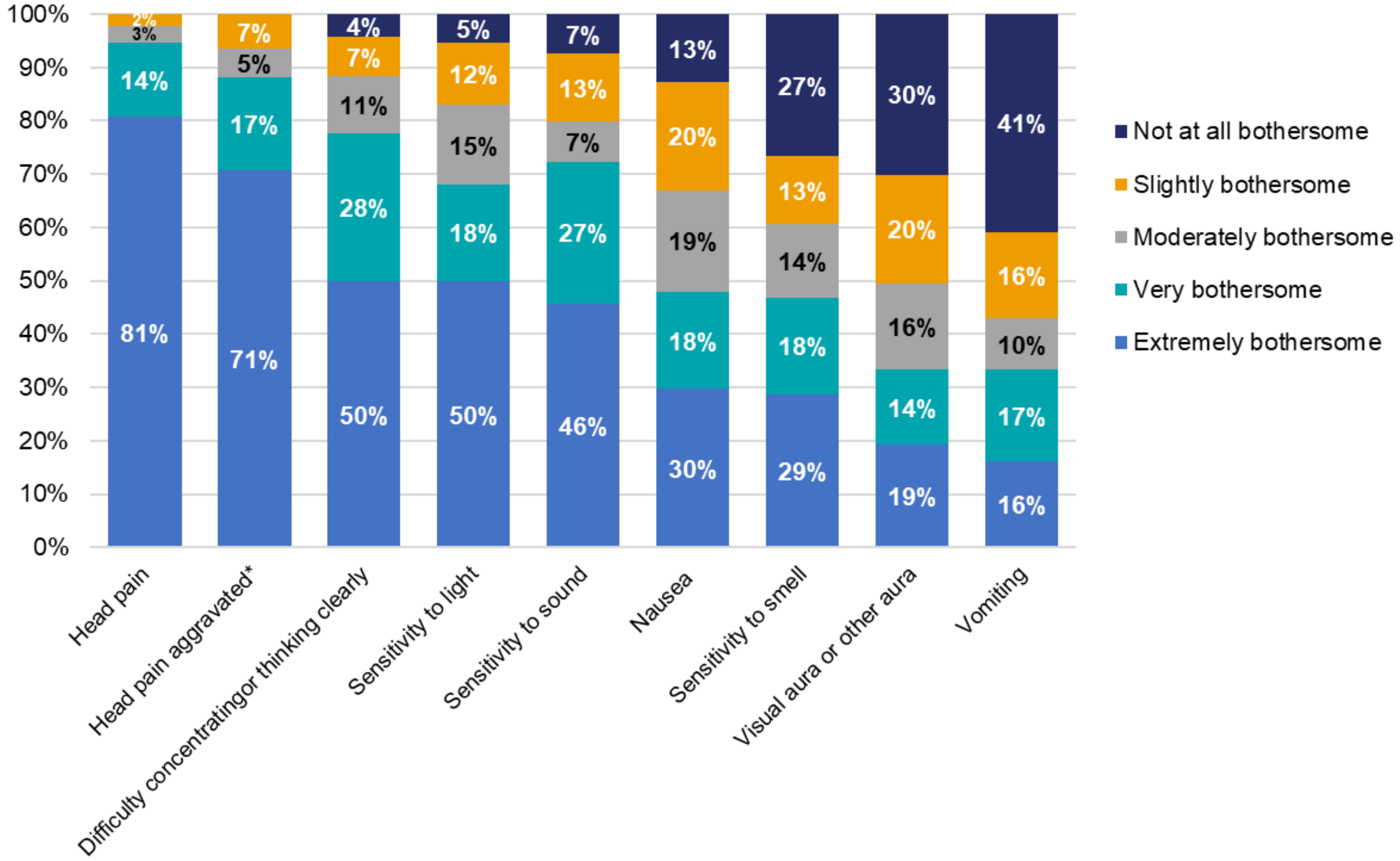

*Head pain that worsened with any movement, or routine physical activity

Patients were instructed: “Please indicate (check ✓) how bothersome you found the following symptoms (before starting on preventive treatment).” Choices included: not at all bothersome, slightly bothersome, moderately bothersome, very bothersome, and extremely bothersome.
