# Supplementary figures and images for: Real-world effectiveness and satisfaction with intravenous eptinezumab treatment in patients with chronic migraine: REVIEW, an observational, multi-site, US-based study
Source: J Headache Pain. 2024 Apr 25;25(1):65. doi: 10.1186/s10194-024-01764-9 (PMC11044317; doi:10.1186/s10194-024-01764-9)

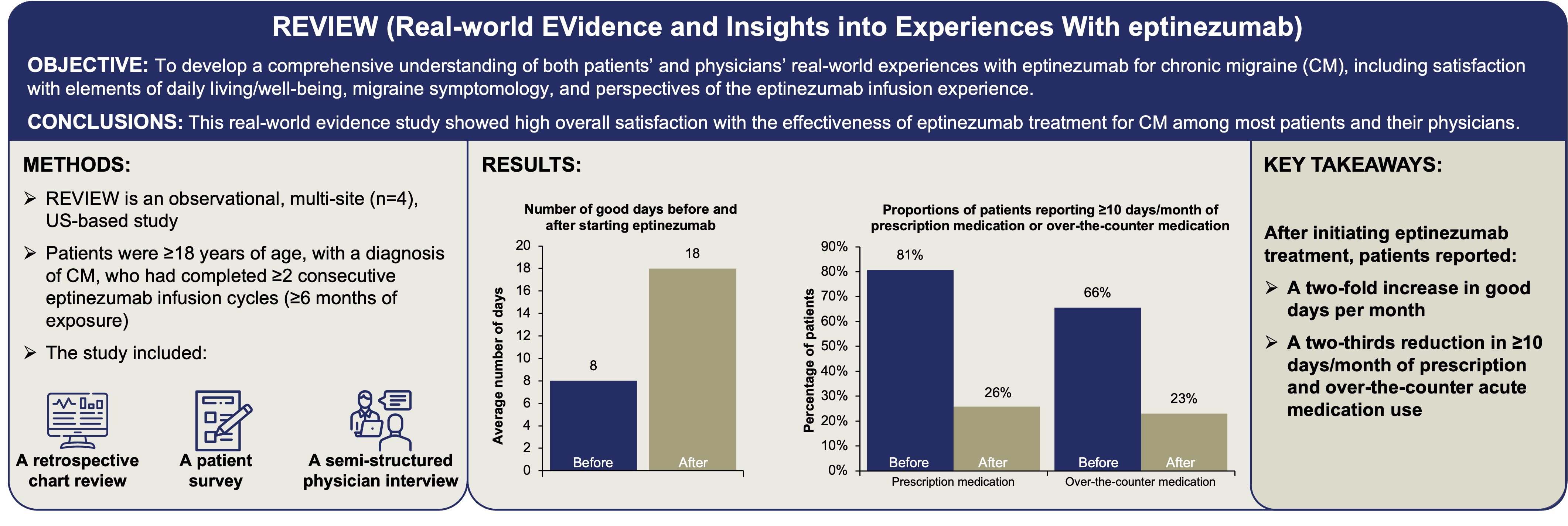

Supplement: Supplementary file 2 — Supplementary Material 2 [file 10194_2024_1764_MOESM2_ESM.jpg]
